# GPT-4 improves sex-specificity in cardiovascular patient education but may perpetuate gender biases: A mixed-methods audit

**DOI:** 10.1371/journal.pdig.0001122

**Published:** 2026-09-10

**Authors:** Samah Khan, Georgeta Vaidean

**Affiliations:** 1 School of Global Public Health, New York University, New York, New York, United States of America; 2 Herbert Wertheim College of Medicine, Florida International University, Miami, Florida, United States of America; Ben-Gurion University of the Negev, ISRAEL

## Abstract

Patient education materials for cardiovascular disease (CVD) prevention frequently omit clinically important differences in disease manifestation, risk, and prevention between sexes. Socially constructed gender norms further shape how health information is communicated and received. Large Language Models (LLMs) like GPT-4 offer a potential tool for personalizing health communication, but their capacity to incorporate evidence-based sex differences without introducing or perpetuating gender biases is unknown. We identified seven publicly available English-language CVD prevention handouts from major health organizations. Using the GPT-4 API in August 2025, we generated sex-specific revisions for a 55-year-old male and female audience via standardized prompts. We provided structured prompts instructing the model to include evidence-based, sex-specific risk factors and symptoms. Original and revised materials were evaluated using Flesch-Kincaid Reading Ease, a novel 10-point sex-inclusivity checklist, and qualitative thematic analysis using a reflexive approach informed by thematic analysis principles. GPT-4 revisions substantially improved sex-inclusivity scores (Original median: 3.0/10, IQR = 0.0–3.0; Male-tailored median: 7.0, IQR = 7.0–8.5; Female-tailored median: 10.0, IQR = 10.0–10.0). Readability was maintained. However, qualitative analysis identified instances where outputs introduced content inconsistent with an equity-oriented framework: female-tailored content occasionally framed female symptoms as deviations from a male norm, male-tailored content frequently omitted erectile dysfunction as a CVD risk marker despite explicit prompting, and both versions at times reflected gendered social stereotypes (e.g., “bottling up emotions”) rather than clinical evidence. LLMs can rapidly improve sex-specificity in patient education materials but may also reproduce gender stereotypes and linguistically biased framings present in their training data. Their integration into clinical content development requires critical human oversight to support, rather than undermine, health equity goals.

## Author summary

**Why was this study done?:**

- Heart disease affects men and women differently, but most patient education materials tend to be the same for everyone. We wanted to see if Artificial Intelligence, specifically large language models like GPT-4, could help rewrite these materials to be more accurate and helpful for each sex.

**What did the researchers do and find?:**

- We took seven heart disease prevention online handouts from major public health and clinical organizations. We used GPT-4 to create new versions specifically for men and for women, including male-specific risk markers (e.g., erectile dysfunction) and female-specific risk enhancers (e.g., menopause). We found that the original handouts were not very sex-specific. The AI-revised versions were substantially better, especially for women, providing more relevant information without making the text harder to read. However, we also found the AI sometimes reflected gendered social assumptions, such as using stereotypes about how men handle emotions rather than citing clinical evidence.

**What do these findings mean?:**

- AI can be a powerful assistant for creating drafts, but it is not a replacement for human expertise. To be used safely, a healthcare professional must always review AI-generated content to ensure it is medically accurate and free from harmful stereotypes.

## Introduction

Cardiovascular disease (CVD) remains the foremost cause of mortality globally, yet its manifestation, risk profiles, and optimal prevention strategies exhibit significant variation between sexes [1,2]. For instance, women are more likely to present with presentations that differ from the classically described chest pain, with symptoms such as fatigue, nausea, or back pain during a myocardial infarction. This often leads to delays in diagnosis and treatment [3,4]. Biological factors, including the cardioprotective role of endogenous estrogen pre-menopause and its decline thereafter, fundamentally alter women's CVD risk trajectory [5]. Conversely, men often develop CVD at a younger age and face distinct risk markers, such as erectile dysfunction [6].

Despite these well-documented differences, a substantial portion of patient-facing educational materials on CVD prevention remains gender-neutral [7]. This “one-size-fits-all” approach, while simplifying content creation, risks flattening critical nuances, potentially leaving patients ill-informed about their specific risks and symptoms. The field of health literacy has long advocated for tailored communication to improve comprehension and self-efficacy [8], yet creating multiple versions of materials is a resource-intensive process.

The emergence of Large Language Models (LLMs) like GPT-4 offers a potential paradigm shift. These models can generate human-like text and follow complex instructions, presenting an opportunity to automate the personalization of health information at scale [9]. A growing body of research has documented that LLMs exhibit significant gender biases across a range of domains [10,11], with studies demonstrating that these patterns persist across multiple state-of-the-art models despite active debiasing efforts [12]. Emerging work has begun to examine these patterns specifically in clinical contexts, including cardiovascular care [13] and patient-facing health content [14]. Studies have explored LLMs for improving readability and simplifying medical jargon, and recent work has evaluated LLM-generated content for accuracy and health literacy adequacy [15]. However, systematic evaluation of LLMs’ capacity to integrate evidence-based sex differences into patient-facing materials without reproducing gendered social stereotypes or linguistically biased framings remains limited, particularly in the domain of cardiovascular disease prevention.

We hypothesized that GPT-4 can be strategically prompted to revise existing CVD prevention materials into sex-specific versions that are both highly readable and significantly more inclusive of evidence-based sex differences. Specifically, we aimed to: (1) quantify the extent of sex-specific content in widely used patient education handouts, and (2) evaluate the efficacy of GPT-4 in enhancing sex-specific representation while preserving or improving readability. In addition to assessing quantitative performance, we examined whether the revision process introduced gendered social stereotypes or linguistically biased framings inconsistent with equity-oriented health communication. Our study therefore serves as a critical, real-world evaluation of a prominent AI tool for a core digital health application: the scalable personalization of patient-facing communication.

## Results

### 1. Quantitative Improvements in sex-specificity and readability

The seven original patient education handouts demonstrated moderate readability (Median Flesch Reading Ease = 64.0, IQR = 60.7–71.2). However, their performance on the sex-inclusivity checklist was poor (Median Score = 3.0, IQR = 0.0–3.0) (Table 1). Notably, materials from the CDC and NHS scored 0/10, containing no sex-specific content. MedLine Plus scored highest (6/10) but still omitted key differentiators like symptom variation. No original material explicitly discussed hormonal influences like testosterone's role in male cardiovascular health or pregnancy-related risks for women.

**Table 1:** Readability and Sex-Inclusivity Scores for Original and GPT-4 Revised Patient Education Materials. *Mayo Clinic Male-Tailored score corrected from originally reported value of 9–6 following second-reviewer scoring reconciliation.

Source	Orig FRE	Male FRE	Female FRE	Orig Score	Male Score	Female Score
Mayo Clinic	71.4	73.2	73.6	3	6*	10
AHA	58.5	68.8	70.3	2	7	10
Cleveland Clinic	68.7	73.5	67.8	3	8	10
CDC	63	62.5	65.1	0	9	10
NHS	60.7	71.4	75.9	0	7	9
UC Davis	64	78.8	68.1	3	7	10
MedLine Plus	71.2	75.6	73.8	6	9	10
**Median (IQR)**	64.0 (60.7–71.2)	73.2 (68.8–75.6)	70.3 (67.8–73.8)	3.0 (0.0–3.0)	7.0 (7.0–8.5)	10.0 (10.0–10.0)

**Fig 1:**
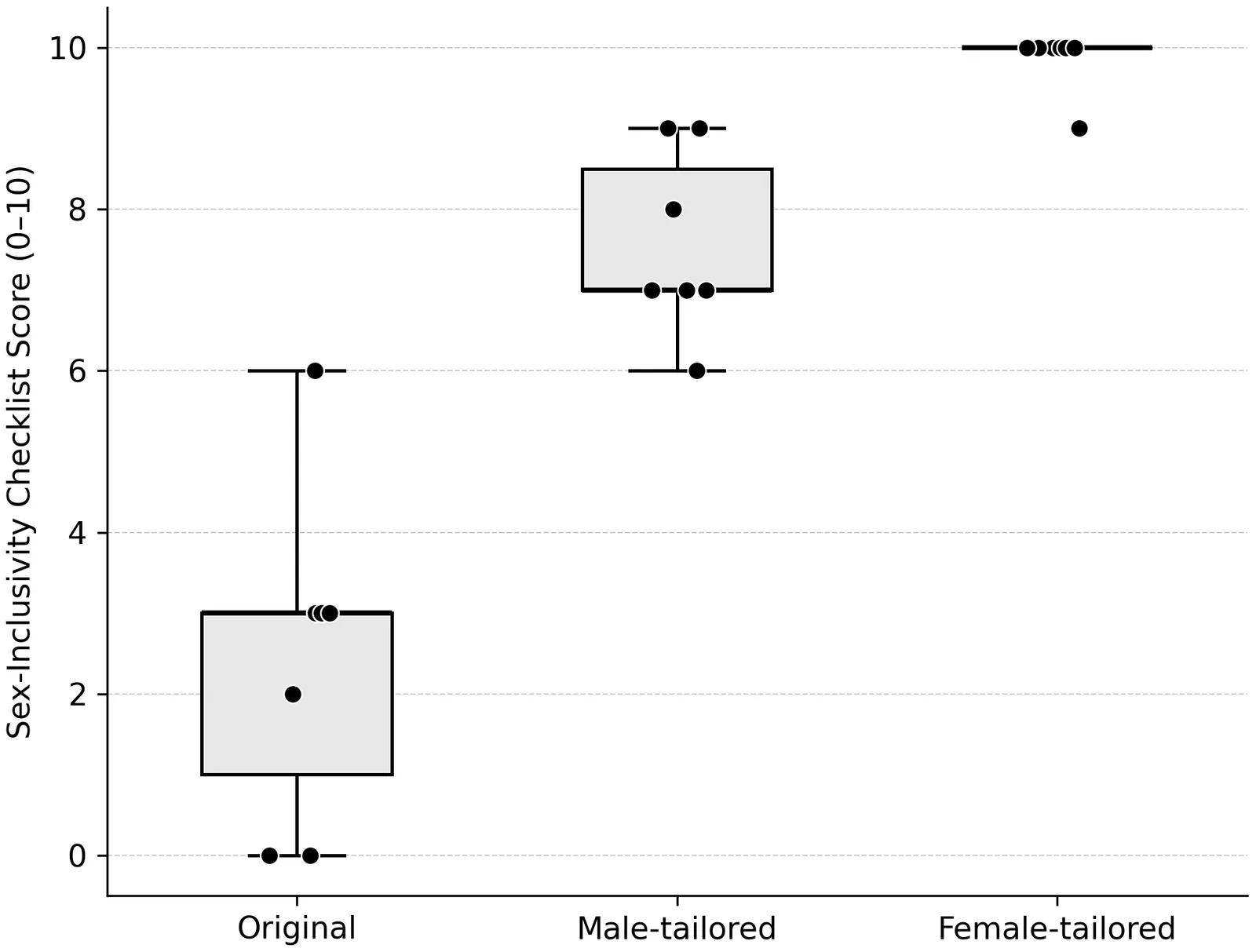
Sex-Inclusivity Checklist Scores by Revision Condition. Box plots show the distribution of sex-inclusivity checklist scores (0-10) for the seven CVD prevention handouts in their original form and after GPT-4 revision for a male-tailored and female-tailored audience. Individual source scores are overlaid as points. Boxes represent the interquartile range with the median indicated by the horizontal line; whiskers extend to the minimum and maximum observed values.

GPT-4 revision led to a dramatic and consistent improvement in sex-inclusivity scores for both male- and female-tailored versions (Fig 1). The increase was most pronounced for female-tailored materials, which achieved a perfect median score of 10.0 (IQR = 10.0–10.0). All seven female-tailored versions addressed female-predominant symptoms, menopause-related risk, and hormonal influences. Male-tailored versions also showed significant improvement (Median Score = 7.0, IQR = 7.0–8.5), though they occasionally omitted specific factors like erectile dysfunction.

A Friedman test revealed a statistically significant difference in sex-inclusivity scores across the three conditions (χ²(2) = 14.0, p < 0.001). Post-hoc Nemenyi tests confirmed that female-tailored scores were significantly higher than original scores (p < 0.001). The difference between original and male-tailored scores did not reach statistical significance (p = 0.147), nor did the difference between male- and female-tailored scores (p = 0.147).

GPT-4 revisions not only enhanced sex-specificity but also maintained high readability. The median Flesch Reading Ease score was 73.2 (IQR = 68.8–75.6) for male-tailored and 70.3 (IQR = 67.8–73.8) for female-tailored versions. A separate Friedman test found no statistically significant difference in readability scores across the three conditions (χ²(2) = 5.43, p = 0.066), indicating that the process of adding complex, specific information did not compromise textual accessibility.

### 2. Qualitative analysis reveals instances of gender stereotyping and linguistic bias

Qualitative thematic analysis of the GPT-4 outputs identified several instances in which outputs reflected gendered social assumptions or linguistically biased framings, despite the structured prompting intended to constrain outputs to clinically established content. These instances, while limited in number, are substantively meaningful in a patient-facing health communication context.

**Linguistic framing of male-as-default:** In describing symptoms, the NHS female-tailored revision noted that women experience “less common” heart attack symptoms. This framing positions the male clinical presentation as the norm and the female presentation as a deviation. This pattern is consistent with the androcentric bias historically prevalent in medicine, with documented consequences for diagnostic delay in women [16]. Notably, this framing emerged without instruction; it was an unrequested artifact of the model's generation.

**Omission of male-specific biology:** Despite explicit prompting, four of seven male-tailored revisions failed to mention erectile dysfunction as a CVD risk marker, and most did not address the role of testosterone. Whether this reflects underrepresentation in training data, prompt interpretation, or model-level content prioritization cannot be determined from the current study design.

**Reinforcement of gendered social stereotypes:** The model occasionally substituted clinical content with socially constructed generalizations. For example, one male-tailored revision stated, “Men are more likely to bottle up emotions, which can lead to stress-related health problems,” which is a non-evidence-based social trope that replaces biomedical content.

**Asymmetric gendered framing of stress across multiple outputs:** A recurring pattern across three female-tailored revisions involved framing women as inherently more stress-prone, without clinical citation. The Mayo Clinic female revision stated that “women tend to feel more stress than men”; the MedLine Plus female revision stated that “women may feel stress more intensely”; and the UC Davis female revision noted that “chronic stress is linked to heart disease, and women are often more affected by stress.” This pattern is notable both for its consistency across independent outputs and for the absence of equivalent framing in the corresponding male-tailored revisions, where stress was addressed as a general modifiable risk factor without sex-specific characterization. As with the “bottling up emotions” example in the male-tailored content, these statements substitute gendered social assumptions for clinical evidence.

**Gendered assumptions in preventive actions:** Recommendations for physical activity and overcoming barriers at times reflected traditional gender roles. For example, in the Mayo Clinic revision, the term ‘housekeeping’ as an example of physical activity was retained only in the female-tailored version, and ‘gardening,’ which was also present in the original, was similarly preserved only in the female-tailored revision. The male-tailored version retained only non-domestic examples such as ‘walking the dog’ and ‘taking the stairs.’ A male-tailored revision of a different source (Cleveland Clinic) separately suggested ‘affordable gyms’ as a resource for overcoming barriers to exercise. These patterns illustrate how the model selectively mapped domestic activity examples onto female content and gym-based framing onto male content. A related clinical framing difference was observed in the UC Davis revisions: the female-tailored version noted that women “need to be careful with aspirin because it can sometimes cause stomach issues,” while the male-tailored version recommended aspirin without qualification. While aspirin recommendations do differ by sex in current clinical guidelines, the female framing emphasized GI risk without addressing the distinct risk-benefit calculus that underlies sex-differentiated aspirin guidance, potentially reducing a nuanced clinical distinction to a cautionary note about side effects.

## Discussion

### Principal findings

Our study suggests that GPT-4 was highly effective at integrating evidence-based, sex-specific information into CVD prevention materials, with a particularly strong performance in creating content for women. This finding is consistent with prior documentation of gender disparities in cardiovascular patient education [7] and aligns with broader observations that LLMs may reflect the distribution of topics in their training corpora [10]. The stronger performance for female-tailored content likely reflects increased volume of public health literature addressing the historical underrepresentation of women in cardiovascular research and clinical guidelines [2], which may be more prominently represented in the model's training data. The improvement in male-tailored content, while statistically significant at the overall Friedman level, was less complete: four of seven revisions omitted erectile dysfunction as a CVD risk marker despite explicit prompting, suggesting that nuanced men's health topics may be less consistently represented in the model's training corpus or de-emphasized in clinical communication literature relative to female-specific content.

### Implications of bias and stereotype perpetuation

An important finding of this study is that quantitative improvements in sex-specific content inclusion do not necessarily guarantee equity in the framing and tone of that content. While GPT-4 successfully added clinically relevant information in the majority of outputs, qualitative analysis identified several instances in which outputs reflected patterns consistent with gender stereotypes or linguistically biased framings plausibly derived from training data. These findings, while based on a limited number of identified instances across 14 total generations, raise substantive concerns warranting attention in the context of patient-facing health communication.

The omission of erectile dysfunction as a CVD risk marker in four of seven male-tailored revisions, despite explicit prompting, represents a notable gap in clinical completeness. The poor inter-rater reliability observed for male-tailored materials (weighted κ ranging from −0.06 to 0.19) may reflect genuine ambiguity in male-specific CVD content, a pattern consistent with this qualitative finding. The use of social generalizations such as “bottling up emotions” in place of evidence-based content suggests the model may draw on culturally dominant narratives about gender when direct clinical evidence is sparse or ambiguous. This pattern was not isolated to a single output. Three female-tailored revisions independently characterized women as more emotionally reactive to stress than men, which is a framing unsupported by the clinical CVD literature and consistent with documented gender stereotyping tendencies in LLMs [10,11]. The convergence of this pattern across multiple independent generations strengthens the case that it reflects a systematic tendency in the model's outputs rather than a one-off artifact.

The linguistic framing of female CVD symptoms as “less common” rather than simply “different” reflects a male-as-default paradigm with documented consequences for diagnostic delay in women [3,16]. This framing was identified in only one output (NHS female revision), but its presence is notable given that the prompt did not instruct the model to use such framing; it emerged as an unrequested artifact of generation. Together, these observations suggest that LLMs may, in some contexts, reproduce or introduce biased framings beyond simply reflecting statistical distributions in their training data, though the present study is not designed to adjudicate this mechanistic question.

These findings are consistent with a growing body of empirical evidence demonstrating that gender biases in LLMs persist across multiple state-of-the-art models despite active debiasing efforts [12], and that GPT-4 specifically has been shown to perpetuate racial and gender biases in clinical contexts including diagnostic reasoning and patient assessment [13]. They further align with parallel work demonstrating that LLM outputs in psychiatric care showed significant racial bias in treatment recommendations despite relatively consistent diagnostic reasoning, a pattern in which surface-level performance metrics obscured equity-relevant failures in output quality [14]. These findings underscore the necessity of integrating qualitative and equity-focused evaluation frameworks alongside quantitative metrics in LLM health communication research.

### Limitations and future directions

This study has several limitations. The sample size of seven handouts, while diverse in source, is small, limiting the generalizability of both quantitative and qualitative findings. The sex-inclusivity checklist was developed de novo for this study, informed by established literature; while its domains reflect consensus clinical content, it has not been externally validated. The evaluation of sex-inclusivity scores relied primarily on a single reviewer; while a second independent rater was engaged to assess all 21 materials (see Methods), the limited sample size constrains the generalizability of the inter-rater reliability estimate. The qualitative analysis was conducted by a single primary analyst, which, while consistent with reflexive thematic analysis methodology, introduces the possibility of analyst influence; triangulation through co-author review was used to partially mitigate this.

The qualitative findings are based on a relatively small number of identified instances across 14 total generations and should be interpreted as illustrative of the types of bias that may emerge rather than characterizing the frequency or severity of such patterns at scale. Additionally, the study did not include an explicit evaluation of factual correctness of AI-generated outputs beyond checklist domain scores; a clinical expert review assessing the accuracy of specific claims would strengthen future work. We also did not include patient perspectives on the perceived empathy, trustworthiness, or usefulness of the materials, nor test comprehension or behavior change, which are both vital next steps.

Future research should explore sex- and gender-aware prompting strategies that explicitly instruct the model to avoid social stereotypes and use patient-centric language; evaluate materials with diverse patient groups; extend the approach to non-binary individuals; adapt materials into other languages; and apply the framework to other disease areas. Finally, this study evaluated a single LLM (GPT-4) using a specific prompting approach at a fixed temperature setting. The extent to which these findings generalize to other contemporary LLMs, model versions, or alternative prompting strategies remains unknown and warrants investigation in future work.

### Conclusion

LLMs like GPT-4 represent a powerful but double-edged tool for creating equitable health education. They can rapidly generate more sex-specific content but may also reproduce gendered social stereotypes and linguistically biased framings. Therefore, their implementation must be guided by a framework of critical digital health literacy, where AI assists in drafting, but human expertise, particularly in ethics and health equity, remains essential for editing, validation, and ensuring the promotion of authentic patient-centered care.

## Materials and methods

### 1. Material selection

To simulate a patient's information-seeking behavior, we conducted a systematic search using the Google search engine on August 17, 2025. To maximize reproducibility and minimize personalization effects, the search was conducted using Google Chrome in Incognito mode, with geographic location set to the United States, language settings configured to English, and no active Google account sign-in. The search query ‘how to prevent a heart attack’ was entered verbatim. We included the top 7 unique English-language patient education handouts from the first page of results, representing major public health and clinical organizations (e.g., CDC, NHS, Mayo Clinic). Sponsored results and non-handout content (e.g., news articles, homepages) were excluded. This approach was chosen to capture the materials a typical patient is most likely to encounter during common information-seeking behavior, thereby enhancing the external validity of our study. All original handouts are available in the OSF repository.

### 2. Terminology

Throughout this manuscript, we use “sex” to refer to biological differences (hormonal, anatomical, and physiological) that inform CVD risk, symptoms, and prevention. We use “gender” to refer to socially constructed norms, roles, and expectations that shape how health information is communicated and received. Where findings reflect both the checklist scores (which assess biological sex-specific content) and the qualitative analysis (which identifies socially constructed gender stereotypes), we specify the relevant dimension. This distinction is maintained consistently throughout.

### 3. LLM revision process

Each handout was processed using the OpenAI GPT-4 API on August 17, 2025 with the specified prompts. The chat completion API was used in a temporary (stateless) session to ensure that each prompt was processed independently without influence from previous interactions. The phrase ‘where clinically established’ was included in the prompts to constrain the model's output to only those sex-specific differences supported by robust clinical evidence, thereby minimizing the generation of unverified or speculative content. We used the GPT-4 model with a temperature setting of 0 to maximize determinism and reproducibility. Prompts were refined through an iterative piloting process with three handouts to establish reliability. These pilot handouts were not included in the final analysis of the seven primary handouts. All outputs were saved verbatim.

**Prompt for male-tailored revision:**

“Revise this heart disease prevention material for a 55-year-old male audience at a 6th-grade reading level. Where clinically established, explicitly include: (1) Male-predominant symptom presentations; (2) Biological risk factors more common in men; (3) Prevention strategies with different efficacy/considerations for men; (4) Maintain strict medical accuracy and cite only evidence-based differences.”

**Prompt for female-tailored revision:**

“Revise this heart disease prevention material for a 55-year-old female audience at a 6th-grade reading level. Where clinically established, explicitly include: (1) Female-predominant symptom presentations; (2) Biological risk factors more common in women; (3) Prevention strategies with different efficacy/considerations for women; (4) Maintain strict medical accuracy and cite only evidence-based differences.”

### 4. Evaluation metrics

A novel 10-point sex-inclusivity checklist was developed de novo based on a comprehensive review of literature on sex-based differences in CVD [17–22]. It consisted of five domains: (1) sex-specific symptoms, (2) biological risk factors, (3) hormonal influences, (4) sex-specific risk markers (e.g., erectile dysfunction), and (5) considerations for prevention strategies. Each domain was scored 0 (absent), 1 (mentioned but incomplete), or 2 (clearly and accurately described), for a total of 10 points. The checklist was designed to capture biologically grounded sex differences; gendered social patterns identified in the outputs were assessed separately through qualitative analysis (see below). The choice of a 0–2 scale per domain reflects the binary-to-partial-to-complete presence of specific clinical content items; this scale was considered appropriate given the structured, criteria-based nature of the domains, where the primary distinction is between absence, partial mention, and accurate description.

**Quantitative scoring:** Domain 5 (Considerations for Prevention Strategies) was excluded from the inter-rater reliability assessment as it assesses contextual and communicative factors rather than biologically grounded sex-specific clinical content. Inter-rater reliability was assessed using linearly weighted Cohen's kappa per domain, reported separately by condition. For original materials, weighted kappa ranged from 0.59 to 0.65, with exact agreement ranging from 71.4% to 85.7%, indicating moderate to substantial agreement. For female-tailored materials, kappa was not computable due to near-perfect agreement; observed agreement ranged from 57.1% to 100%. For male-tailored materials, weighted kappa ranged from -0.06 to 0.19, with exact agreement ranging from 28.6% to 57.1%, indicating poor inter-rater agreement.

**Qualitative analysis:** Qualitative analysis of the 14 GPT-4-generated outputs was conducted using a reflexive thematic analysis approach, drawing on the framework described by Braun and Clarke [23]. This approach was selected for its flexibility in capturing both semantic (explicit) and latent (interpretive) content in text. The primary analyst (S.K.) conducted multiple readings of all outputs, generating initial codes that captured both the presence and framing of sex-specific and gendered content. Codes were then organized into candidate themes, reviewed and refined iteratively, and finalized through discussion between both authors. Analysis was oriented by two conceptual lenses specified prior to coding: (1) the accuracy and completeness of biological sex-specific information relative to current clinical evidence, and (2) the presence of socially constructed gender norms or stereotypes in language and recommendations. The distinction between biological sex differences and gendered social constructs was preserved throughout reporting. Analyst triangulation was achieved through iterative discussion and review of coded excerpts by both authors; disagreements were resolved through consensus.

### 5. Data analysis

Descriptive statistics (medians and interquartile ranges) were used to summarize readability and sex-inclusivity scores. Given the small sample size (N = 7 handouts) and the ordinal nature of the checklist scores, the non-parametric Friedman test was used to compare sex-inclusivity scores across the three revision types (Original, Male-tailored, Female-tailored). A Friedman test was also performed on the Flesch Reading Ease scores. A statistically significant Friedman test was followed by post-hoc pairwise comparisons (Nemenyi test). Statistical analysis was performed using StatsKingdom.com. A p-value of <0.05 was considered statistically significant.
