# Supplementary material for: GPT-4 improves sex-specificity in cardiovascular patient education but may perpetuate gender biases: A mixed-methods audit
Source: PLOS Digit Health. 2026 Sep 10;5(9):e0001122. doi: 10.1371/journal.pdig.0001122 (PMC13561376; doi:10.1371/journal.pdig.0001122)
Supplement: Supplementary file 1 [file pdig.0001122.s002.docx]

**RESPONSE TO REVIEWERS**

**Manuscript:** GPT-4 Improves Sex-Specificity in Cardiovascular Patient Education but May Perpetuate Gender Biases: A Mixed-Methods Audit

**Manuscript Number:** PDIG-D-25-01045R1

We thank the editors and all three reviewers for their thoughtful and constructive feedback. The comments have substantially strengthened the manuscript. Below we respond to each point in turn. All changes are reflected in the tracked-changes version of the revised manuscript.

# **EDITOR COMMENTS**

## **Editor Comment 1: Sex vs. gender terminology**

*"The manuscript needs clearer definitions and consistent use of 'sex' versus 'gender' terminology, as the analysis conflates biological differences with socially constructed gender norms."*

**Response:** We agree this was a meaningful conceptual gap in the original submission. We have added an explicit terminology section (Materials and Methods, Section 2) defining "sex" as biological attributes including hormonal and physiological differences, and "gender" as socially constructed roles, norms, and identities. This distinction is now maintained consistently throughout the manuscript. The checklist is relabeled as the "sex-inclusivity checklist" to reflect its focus on biological content, while qualitative findings that identify social stereotypes are explicitly framed as "gendered social bias" rather than sex-based differences. The title has also been revised to reflect both dimensions.

**Manuscript change:** New Section 2 (Terminology) added to Methods, terminology updated throughout, title revised.

## **Editor Comment 2: Title and "amplifies" claim**

*"The title and key claims — particularly that GPT-4 'amplifies' gender biases — are not sufficiently supported by the evidence presented."*

**Response:** We agree. The term "amplifies" implies a measured increase over a quantified baseline, which our study design cannot support. The title has been revised to "GPT-4 Improves Sex-Specificity in Cardiovascular Patient Education but May Perpetuate Gender Biases," and all instances of "amplifies," "actively generated and amplified," and related language throughout the manuscript have been replaced with more appropriately hedged formulations (e.g., "may reproduce," "introduced instances of," "reflected patterns consistent with"). The qualitative findings are now explicitly framed as illustrative rather than definitive.

**Manuscript change:** Title revised, language moderated throughout Abstract, Results, Discussion, and Conclusion.

## **Editor Comment 3: Inter-rater reliability**

*"Reliance on a single reviewer for scoring, with no inter-rater reliability assessment."*

**Response:** We have engaged a second independent reviewer (G.V.) to score all 21 materials on Domains 1–4 of the sex-inclusivity checklist. Domain 5 (Tone & Language) was excluded from the inter-rater reliability assessment as it was not consistent with the primary objective of the checklist, which was to assess the presence and completeness of biologically grounded sex-specific clinical content. Inter-rater reliability was assessed using linearly weighted Cohen's kappa per domain, reported separately by condition. For original materials, weighted kappa ranged from 0.59 to 0.65, with exact agreement ranging from 71.4% to 85.7%, indicating moderate to substantial agreement. For female-tailored materials, kappa was not computable due to near-perfect agreement; observed agreement ranged from 57.1% to 100%. For male-tailored materials, weighted kappa ranged from -0.06 to 0.19, with exact agreement ranging from 28.6% to 57.1%, reflecting the genuine ambiguity in male-specific CVD content also documented in the qualitative findings. These results are reported in the updated Methods section (Section 4, Quantitative Scoring).

**Manuscript change:** Inter-rater reliability assessment added to Section 4 (Evaluation Metrics).

## **Editor Comment 4: Qualitative methodology**

*"Lack of explicit qualitative analysis methodology."*

**Response:** We have added an explicit qualitative analysis subsection to the Methods (Section 4), describing our use of reflexive thematic analysis following Braun and Clarke (2006). The section details the analytical process including initial coding, theme development, and the two conceptual lenses used to orient analysis (biological sex-specific accuracy and gendered social stereotyping). The distinction between these two dimensions is preserved throughout reporting.

**Manuscript change:** Qualitative Analysis subsection added to Section 4 (Evaluation Metrics).

## **Editor Comment 5: Scoring scheme justification**

*"Insufficient justification for the 0–2 scoring scheme versus more discriminating scales."*

**Response:** We have added explicit justification for the 0–2 scale in the Methods. The three-point scale was selected because the checklist domains assess the presence, partial inclusion, or complete and accurate inclusion of specific clinical content items. This conceptual structure maps naturally onto three levels. Given the structured, criteria-based nature of the items and the small sample size, a broader scale would introduce artificial precision without improving discriminatory validity. This justification is now stated explicitly in the manuscript.

**Manuscript change:** Scoring justification added to Section 4 (Evaluation Metrics).

## **Editor Comment 6: Literature engagement**

*"Insufficient engagement with existing literature on gender bias in LLMs and patient education materials."*

**Response:** We have substantially expanded the Introduction to engage with the existing literature on gender bias in LLMs. We now cite five new references documenting gender bias in LLMs across domains (Kotek et al., 2023; Gallegos et al., 2024), persistence of bias across models despite debiasing efforts (An et al., 2025), GPT-4 specifically perpetuating racial and gender biases in clinical contexts (Zack et al., 2024), and parallel findings in psychiatric care (Bouguettaya et al., 2025). The Introduction now clearly situates our specific contribution as a structured audit of LLM-generated CVD patient education materials, addressing a gap within this broader, active literature rather than claiming the entire topic is unexplored.

**Manuscript change:** Introduction revised, five new references added (refs 18–22).

## **Editor Comment 7: Google search reproducibility**

*"Unclear Google search conditions for reproducibility."*

**Response:** We have added explicit detail on search conditions to the Material Selection section, including use of Chrome Incognito mode, US geographic location, English language settings, and no active Google account sign-in.

**Manuscript change:** Section 1 (Material Selection) updated with search condition details.

## **Editor Comment 8: Data availability**

*"We have been unable to access the data using the link/accession number provided."*

**Response:** We thank the editors for flagging this. The DOI linked to the project registration rather than the project files directly. We have updated the Data Availability Statement to link directly to the project repository (https://osf.io/8skgh), where all original handouts, GPT-4 outputs, and analysis materials are accessible and organized by folder.

**Manuscript change:** Data Availability Statement updated with direct repository link.

# **REVIEWER 1 COMMENTS**

## **Comment 1: Define readability**

*"Please include a definition for 'readability' in terms of how you are measuring the hypothesized 'highly readable' GPT outputs."*

**Response:** Thank you for this suggestion. We have added a definition of the Flesch-Kincaid Reading Ease score to the Introduction, specifying that it is a validated metric in which higher scores indicate greater textual accessibility and scores of 60–70 correspond to standard newspaper-level comprehension.

**Manuscript change:** Readability definition added to Introduction.

## **Comment 2: Label prompts explicitly**

*"Please call out the location of the standardized prompts more explicitly."*

**Response:** The prompts are now presented as explicitly labeled blocks within Section 3 (LLM Revision Process) of the Methods, with the preceding text referencing them directly.

**Manuscript change:** Prompts formatted as labeled blocks in Section 3.

## **Comment 3: Qualitative methodology**

*"Please include methodology for your qualitative analysis."*

**Response:** We have added an explicit qualitative analysis subsection to the Methods (Section 4), describing our use of reflexive thematic analysis following Braun and Clarke (2006). The section details the analytical process including initial coding, theme development, and the two conceptual lenses used to orient analysis (biological sex-specific accuracy and gendered social stereotyping). The distinction between these two dimensions is preserved throughout reporting.

**Manuscript change:** Qualitative Analysis subsection added to Section 4.

## **Comment 4: Situate findings in existing literature**

*"Please present findings more in relation to the existing relevant literature."*

**Response:** We have substantially expanded the Introduction to engage with the existing literature on gender bias in LLMs, citing five new references documenting gender bias across domains (Kotek et al., 2023; Gallegos et al., 2024), persistence of bias despite debiasing efforts (An et al., 2025), GPT-4 perpetuating biases in clinical contexts (Zack et al., 2024), and parallel findings in psychiatric care (Bouguettaya et al., 2025). The Discussion section 3.2 now explicitly situates our qualitative findings within this broader body of work.

**Manuscript change:** Introduction and Discussion revised with new citations.

# **REVIEWER 2 COMMENTS**

## **Comment 1: Overclaiming and bold statements**

*"The authors use definitive and bold statements throughout the manuscript without backing them up."*

**Response:** We agree this was a significant weakness of the original submission. We have systematically moderated the language throughout by replacing "amplifies" with "may perpetuate," "actively generated and amplified" with "reflected patterns consistent with," and similar reformulations. All major claims are now explicitly hedged to match the strength and scope of the evidence. The qualitative findings are framed as illustrative instances rather than definitive characterizations of model behavior.

**Manuscript change:** Language moderated throughout Abstract, Introduction, Results, Discussion, and Conclusion.

## **Comment 2: Contribution to state of the art**

*"It is unclear what the overall contribution of this paper is to the state of the art."*

**Response:** We have revised the Introduction to more clearly articulate our specific contribution. While a growing literature documents gender bias in LLMs broadly and in clinical contexts, our study addresses a specific and underexplored application: the use of LLMs to personalize patient-facing cardiovascular education materials, evaluated using both a structured quantitative checklist and qualitative thematic analysis. This framing is now explicit in the final paragraph of the Introduction. To our knowledge, no prior study has systematically evaluated LLM-generated patient-facing CVD education materials using both quantitative sex-inclusivity scoring and qualitative bias auditing. This combination reveals how surface-level metric performance can obscure equity-relevant failures in output framing and content.

**Manuscript change:** Introduction revised, contribution statement clarified.

## **Comment 3: "Largely unexplored" claim**

*"The literature on this topic is quite rich, and the authors didn't provide a single reference on what has been explored already."*

**Response:** We no longer claim the topic is largely unexplored. The Introduction has been substantially revised to engage with the existing literature on gender bias in LLMs (Kotek et al., 2023; Gallegos et al., 2024; An et al., 2025) and in clinical AI contexts (Zack et al., 2024; Bouguettaya et al., 2025). We now situate our specific contribution as a structured audit of LLM-generated CVD patient education materials — addressing a gap within this broader, active literature rather than claiming the entire topic is unexplored.

**Manuscript change:** Introduction revised with five new citations.

## **Comment 4: Subjective premise and author bias**

*"The entire premise of the paper is based on a subjective assumption... the authors stated 'we found that the original handouts were not very gender-specific.'"*

**Response:** The characterization of original handouts as lacking sex-specific content is now grounded explicitly in the quantitative checklist scores (Median = 3.0/10, with two materials scoring 0/10) rather than presented as an impressionistic judgment. We have also added a Terminology section that clarifies the conceptual framework underpinning the study, and the qualitative findings are now presented with explicit a priori thematic categories to reduce the appearance of post-hoc interpretation.

**Manuscript change:** Results framing revised, Terminology section added, qualitative methodology section added.

## **Comment 5: "Historical neglect" claim needs citation**

*"Statements such as 'addressing the historical neglect of women's cardiovascular health' must be backed by a reference."*

**Response:** This claim is now supported by citation [2] (Garcia et al., 2016, Circulation Research), which documents the historical underrepresentation of women in cardiovascular research and clinical trials.

**Manuscript change:** Citation added to relevant sentence in Discussion.

## **Comment 6: Amplification claim not backed by evidence**

*"The fact that a specific LLM, under a specific prompt, on a specific task, generates a statement with a perceived bias does not lead to the broad conclusion that GPT-4 actively generated and amplified biases."*

**Response:** We fully agree. This language has been removed. The Discussion now states that outputs "reflected patterns consistent with gender stereotypes or linguistically biased framings plausibly derived from training data" and explicitly notes that "the present study is not designed to adjudicate this mechanistic question." The findings are appropriately scoped to what the study design can support.

**Manuscript change:** Discussion section 3.2 revised.

## **Comment 7: Anecdotal evidence and a priori criteria**

*"From the 14 generations, 4 instances are considered by the authors to include biases. No exact criteria for evaluation are established a priori."*

**Response:** The four thematic categories were derived from the study's conceptual framework (distinguishing biological sex-specific accuracy from gendered social stereotyping) and were specified prior to detailed qualitative coding. All outputs were then systematically reviewed against these pre-specified categories. This process is now explicitly described in the Methods.

Systematic thematic analysis of all 14 outputs revealed a convergent pattern across three independent female-tailored outputs (Mayo Clinic, MedLine Plus, UC Davis): each characterized women as more stress-prone without clinical citation, using near-identical framing across unrelated source documents. This convergence supports a systematic model tendency rather than isolated artifact and is now reported as a distinct theme in Results Section 2. Additional findings including asymmetric framing in exercise recommendations and clinical guidance differences have been integrated into Results Section 2 as distinct themes.

**Manuscript change:** Qualitative Analysis subsection updated with a priori categories, Limitations revised.

## **Comment 8: Confirmation bias in prompt development**

*"It is unclear how the prompt was developed, and the authors 'refined through an iterative piloting.'"*

**Response:** We thank the reviewer for this important point. The iterative piloting process was used exclusively to ensure technical reliability: specifically, to confirm that the prompt was correctly formatted, that outputs were generated as intended, and that the requested reading level was consistently achieved. Pilot handouts were not included in the final analysis. Critically, piloting did not involve modifying prompt content to influence or optimize for bias detection. We acknowledge in the Limitations that any prompt development process introduces the possibility of researcher influence, and future work would benefit from pre-specifying prompt criteria prior to piloting.

**Manuscript change:** Section 3 (LLM Revision Process) clarified, Limitations updated.

## **Comment 9: Missing Section 3**

*"Section 3 seems omitted or skipped."*

**Response:** The Discussion subsections have been renumbered from 4.1–4.4 to 3.1–3.4 to resolve the numbering gap.

**Manuscript change:** Discussion subsection numbering corrected.

# **REVIEWER 3 COMMENTS**

## **Comment 1: Sex vs. gender terminology**

*"The manuscript alternates between 'gender-specific' and 'sex-based differences' without clear distinction."*

**Response:** We have added an explicit Terminology section (Materials and Methods, Section 2) defining "sex" as biological attributes including hormonal and physiological differences, and "gender" as socially constructed roles, norms, and identities. This distinction is now maintained consistently throughout the manuscript. The checklist is relabeled as the "sex-inclusivity checklist" to reflect its focus on biological content, while qualitative findings that identify social stereotypes are explicitly framed as "gendered social bias." The title has also been revised to reflect both dimensions.

**Manuscript change:** Terminology section added, language standardized throughout.

## **Comment 2: Factual correctness of AI outputs**

*"The study does not include an explicit evaluation of factual correctness of the AI-generated outputs."*

**Response:** We acknowledge this limitation explicitly in Section 3.3 (Limitations), noting that the study evaluates inclusion and representation rather than factual accuracy of specific claims, and that a clinical expert review assessing accuracy would strengthen future work. We note that the second independent reviewer (G.V.), a physician with expertise in cardiovascular medicine, reviewed all AI-generated outputs as part of the inter-rater reliability process and did not identify factual inaccuracies in the content domains assessed. We have also clarified in the Methods that the checklist assesses presence and completeness of content domains rather than clinical accuracy of individual statements.

**Manuscript change:** Limitations section updated, Methods clarified.

## **Comment 3: Scoring scheme justification**

*"I recommend that the authors justify their choice of a 0–2 scoring scheme."*

**Response:** We have added explicit justification for the 0–2 scale in the Methods. The three-point scale was selected because the checklist domains assess the presence, partial inclusion, or complete and accurate inclusion of specific clinical content items. This conceptual structure maps naturally onto three levels. Given the structured, criteria-based nature of the items and the small sample size, a broader scale would introduce artificial precision without improving discriminatory validity. This justification is now stated explicitly in the manuscript.

**Manuscript change:** Scoring justification added to Section 4.

## **Comment 4: Inter-rater reliability**

*"All materials were scored by a single reviewer... I recommend that the authors include a second independent rater."*

**Response:** We have engaged a second independent reviewer (G.V.) to score all 21 materials on Domains 1–4 of the sex-inclusivity checklist. Domain 5 (Tone & Language) was excluded from the inter-rater reliability assessment as it was not consistent with the primary objective of the checklist, which was to assess the presence and completeness of biologically grounded sex-specific clinical content. Inter-rater reliability was assessed using linearly weighted Cohen's kappa per domain, reported separately by condition. For original materials, weighted kappa ranged from 0.59 to 0.65, with exact agreement ranging from 71.4% to 85.7%, indicating moderate to substantial agreement. For female-tailored materials, kappa was not computable due to near-perfect agreement; observed agreement ranged from 57.1% to 100%. For male-tailored materials, weighted kappa ranged from -0.06 to 0.19, with exact agreement ranging from 28.6% to 57.1%, reflecting the genuine ambiguity in male-specific CVD content also documented in the qualitative findings. These results are reported in the updated Methods section (Section 4, Quantitative Scoring).

**Manuscript change:** Inter-rater reliability assessment added to Section 4.

## **Comment 5: Google search conditions**

*"Google search results can vary based on geographic location, personalization, device settings, and time."*

**Response:** We have added explicit detail on search conditions to the Material Selection section (Section 1), including use of Google Chrome in Incognito mode, geographic location set to the United States, language settings configured to English, and no active Google account sign-in. These details are now reported to enable replication of the material selection process.

**Manuscript change:** Section 1 updated.

In addition to the revisions described above, we have made minor copyediting changes throughout for clarity and consistency. No substantive changes were made to the core data, statistical analyses, or primary conclusions beyond those detailed in this response.
