# Supplementary material for: GPT-4 improves sex-specificity in cardiovascular patient education but may perpetuate gender biases: A mixed-methods audit
Source: PLOS Digit Health. 2026 Sep 10;5(9):e0001122. doi: 10.1371/journal.pdig.0001122 (PMC13561376; doi:10.1371/journal.pdig.0001122)
Supplement: Supplementary file 2 [file pdig.0001122.s003.docx]

**REVIEWER 3 COMMENTS: ROUND 2**

***COMMENT 1:*** *"The Methods section should be revised to ensure that the sex-inclusivity checklist domains match the deposited checklist on OSF. In the manuscript, Domain 5 is described as 'considerations for prevention strategies,' whereas the checklist appears to define Domain 5 as 'Tone & Language.'"*

**RESPONSE**: We thank the reviewer for identifying this inconsistency. The discrepancy arose because the OSF checklist had not been updated to reflect revisions made during the previous round of review. We have now updated the deposited checklist on OSF to ensure full consistency with the manuscript. Domain 5 is now labeled "Considerations for Prevention Strategies" in both the manuscript and the OSF repository, reflecting its focus on sex-specific barriers to prevention, tailored risk communication, and acknowledgment of sex differences in treatment adherence or response. The rationale for excluding Domain 5 from inter-rater reliability assessment remains consistent with this updated framing, since it assesses contextual and communicative factors rather than biologically grounded clinical content. The inter-rater reliability description has been updated accordingly throughout the Methods section.

**MANUSCRIPT CHANGE**: Domain 5 label updated in Quantitative Scoring section; OSF checklist updated and re-uploaded.

***COMMENT 2****: "The interpretation of the low inter-rater reliability observed for male-tailored materials appears speculative. Weighted kappa values ranging from -0.06 to 0.19 indicate poor agreement but do not establish that the disagreement arose from genuine ambiguity in male-specific CVD content."*

**RESPONSE**: We agree. We have removed the interpretive claim from the Methods section. The sentence now reads descriptively: "For male-tailored materials, weighted kappa ranged from -0.06 to 0.19, with exact agreement ranging from 28.6% to 57.1%, indicating poor inter-rater agreement." A brief interpretive note has been moved to Discussion section 3.2, where we observe that the low reliability for male-tailored materials may reflect genuine ambiguity in male-specific CVD content, consistent with the qualitative finding that male-tailored outputs frequently omitted erectile dysfunction and testosterone despite explicit prompting. This framing is clearly marked as interpretive rather than established.

**MANUSCRIPT CHANGE**: Kappa interpretation removed from Methods; interpretive note added to Discussion section 3.2.

***COMMENT 3****: "Consider adding to the Limitations section that only GPT-4 was evaluated in this study."*

**RESPONSE**: We thank the reviewer for this suggestion. We have added the following sentence to the Limitations section: "Finally, this study evaluated a single LLM (GPT-4) using a specific prompting approach at a fixed temperature setting. The extent to which these findings generalize to other contemporary LLMs, model versions, or alternative prompting strategies remains unknown and warrants investigation in future work."

**MANUSCRIPT CHANGE**: Limitation added to Section 3.3.
