# Supplementary material for: GPT-4 improves sex-specificity in cardiovascular patient education but may perpetuate gender biases: A mixed-methods audit
Source: PLOS Digit Health. 2026 Sep 10;5(9):e0001122. doi: 10.1371/journal.pdig.0001122 (PMC13561376; doi:10.1371/journal.pdig.0001122)
Supplement: Peer review history

# Peer review: Decision Letter 0

**DOI:** 10.1371/journal.pdig.0001122.r001

**Authors:** Mengyu Wang, Daphna Idan

16 Mar 2026

Response to Reviewers Revised Manuscript with Track Changes Manuscript **Journal Requirements:**

1. Thank you for submitting your data to a repository. We have been unable to access the data using the link/accession number you provided. Please contact the repository to ensure that the links and accession numbers are valid. If a link or accession number needs updating, please provide a revised Data Availability Statement including the information.

For further assistance, you may go to: http://journals.plos.org/digitalhealth/s/data-availability

2. Please upload your main article file as a .doc, .docx or .rtf file.

3. If the reviewer comments include a recommendation to cite specific previously published works, please review and evaluate these publications to determine whether they are relevant and should be cited. There is no requirement to cite these works unless the editor has indicated otherwise.

**Additional Editor Comments (if provided):**

**Conceptual and Framing Issues** : The manuscript needs clearer definitions and consistent use of "sex" versus "gender" terminology, as the analysis conflates biological differences with socially constructed gender norms. The title and key claims - particularly that GPT-4 "amplifies" gender biases - are not sufficiently supported by the evidence presented. Bold statements throughout the manuscript must be tempered to match the strength of the data.

**Methodological Limitations** : the study has several methodological concerns: (1) reliance on a single reviewer for scoring, with no inter-rater reliability assessment; (2) lack of explicit qualitative analysis methodology; (3) insufficient justification for the 0–2 scoring scheme versus more discriminating scales; (4) absence of clinical expert review for factual accuracy of AI outputs; (5) limited experimental scope (one LLM, one search phrase); and (6) unclear prompt development process, raising potential confirmation bias concerns. The authors should also clarify Google search conditions for reproducibility and present the standardized prompts more explicitly.

**Literature Engagement** : There is insufficient engagement with existing literature on gender bias in LLMs and patient education materials in general. The claim that this area is "largely unexplored" is not substantiated, and findings should be situated within the broader body of related work.

**Evidence and Conclusions** **Reviewers' Comments:**

**Comments to the Author**

1. Does this manuscript meet PLOS Digital Health’s publication criteria?

Reviewer #1: Yes

Reviewer #2: No

Reviewer #3: Yes

**********

2. Has the statistical analysis been performed appropriately and rigorously?

Reviewer #1: I don't know

Reviewer #2: No

Reviewer #3: Yes

**********

3. Have the authors made all data underlying the findings in their manuscript fully available (please refer to the Data Availability Statement at the start of the manuscript PDF file)?

The PLOS Data policy

Reviewer #1: Yes

Reviewer #2: No

Reviewer #3: Yes

**********

4. Is the manuscript presented in an intelligible fashion and written in standard English?

Reviewer #1: Yes

Reviewer #2: Yes

Reviewer #3: Yes

**********

Reviewer #1: The present study is interesting. I appreciate how the authors present the work as a pathway to advance equity through more effectively tailored patient education materials in the era of AI, while also ensuring human oversight from content experts.

•Introduction. Please include a definition for “readability” in terms of how you are measuring the hypothesized “highly readable” GPT outputs.

•Methods pg 10. Please call out the location of the standardized prompts more explicitly. For example, you could present them in Box A and write “Each handout was processed using the OpenAI GPT-4 API on August 17, 2025 with the specified prompts (Box A).”

•Methods. Please include methodology for your qualitative analysis.

•Please present findings more in relation to the existing relevant literature (gender-bias in patient education materials and LLM exacerbation of gender bias).

Reviewer #2: This paper explores the use of LLMs such as ChatGPT to develop educational content. The manuscript could be improved as follows:

Major Corrections

- The authors use definitive and bold statements throughout the manuscript without backing them up. The title, emphasizing that the LLMs "amplifies gender biases", is a good example. The authors never demonstrate the amplification of these gender biases, offering only anecdotal evidence from a qualitative analysis of responses. The tone should be modulated with the strength of the data backing it.

- It is unclear what the overall contribution of this paper is to the state of the art, and how this analysis is different from any other analysis on gender biases of LLMs in the literature.

- In line with the previous comment, the authors state that LLMs "ability to address nuanced issues of gender representation and equity in clinical content remains largely unexplored". However, the literature on this topic is quite rich, and the authors didn't provide a single reference on what has been explored already in the literature.

- The author's own biases seem to have permeated the manuscript. The entire premise of the paper is based on a subjective assumption, as the authors stated, "we found that the original handouts were not very gender-specific." Furthermore, the premise of the paper is that materials from the CMC and the NHS "contain no sex-specific content", without delving into why this might be the case.

- Statements such as "addressing the historical neglect of women's cardiovascular health", however accurate, must be backed by a reference in the literature.

- One of the key findings from the paper, such as the LLMs amplify biases, is not backed by substantial evidence. The fact that a specific LLM, under a specific prompt, on a specific task, generates a statement with a perceived bias does not lead to the broad conclusion that "GPT-4 did not merely reflect biases present in its training data; it actively generated and amplified them".

- The overall evidence presented seems anecdotical at best. From the 14 generations (7 handouts, 2 sexes), and based on the qualitative analysis, 4 instances are considered by the authors to include biases. No exact criteria for evaluation is establish a priori, leading to potential confirmation bias.

- The experimental setup is also potentially impacted by confirmation bias. It is unclear how the prompt was developed, and the authors "refined through an iterative piloting". It is unclear what metrics where used to determine the best version of the prompt, or what the authors were optimizing for.

Minor Corrections

- Section 3 seems omitted or skipped.

Reviewer #3: This manuscript addresses an important and timely issue in digital health: whether large language models can personalize cardiovascular patient education materials while avoiding reinforcement of gender bias.

To further strengthen the manuscript, I offer the following comments:

1. The manuscript alternates between the terms “gender-specific” and “sex-based differences” but the operationalization in the Methods suggests that the evaluation focuses primarily on biological differences. At the same time, the qualitative findings identify issues that are distinctly gendered (i.e., socially constructed roles and stereotypes), such as “bottling up emotions,” housekeeping with women and gyms with men. These examples relate to gender norms rather than biological sex.

I recommend that the authors explicitly state the conceptual focus of the study, clearly define how “sex” and “gender” are used, and standardize terminology consistently throughout the manuscript; stereotype (“bottling up emotions”, housekeeping vs gyms) should be called as "gendered social bias".

2. While the prompts instructed GPT-4 to “maintain strict medical accuracy” and include only evidence-based differences, the study does not include an explicit evaluation of factual correctness of the AI-generated outputs. I recommend that the authors consider incorporating a clinical expert review or structured audit to assess factual correctness, omissions, and potential overgeneralizations. If this is not feasible, the conclusions should more clearly acknowledge that the study evaluates inclusion and representation.

3. The manuscript uses a 10-point gender-inclusivity checklist with each domain scored 0–2. In other areas of instrument development, broader rating scales (e.g., 5-point Likert scales) are often used in content evaluation to capture gradations of presence and emphasis, and to improve discriminatory power when quantifying qualitative features. I recommend that the authors justify their choice of a 0–2 scoring scheme relative to alternatives such as a 5-point scale, or discuss whether a broader scale might improve measurement sensitivity for gender-inclusive content.

4. The 10-point gender-inclusivity checklist is central to the quantitative findings; however, all materials were scored by a single reviewer. Distinctions such as “mentioned but incomplete” versus “clearly and accurately described” inherently involve interpretive judgment. I recommend that the authors either include a second independent rater and report inter-rater reliability, or provide more detailed scoring criteria and illustrative examples for each domain. The materials used in the study should be uploaded as supplementary material.

5. The decision to select the top seven results from a Google search enhances ecological validity; however, Google search results can vary based on geographic location, personalization, device settings, and time. To improve reproducibility, I recommend that the authors clarify the search conditions (e.g., use of incognito mode, geographic location, language settings) and provide additional details necessary to replicate the material selection process.

**********

what does this mean?). If published, this will include your full peer review and any attached files.

**Do you want your identity to be public for this peer review?**  If you choose “no”, your identity will remain anonymous but your review may still be made public.

For information about this choice, including consent withdrawal, please see our Privacy Policy

Reviewer #1: No

Reviewer #2: No

Reviewer #3: No

**********

**Figure resubmission:**

**Reproducibility:**

To enhance the reproducibility of your results, we recommend that authors of applicable studies deposit laboratory protocols in protocols.io, where a protocol can be assigned its own identifier (DOI) such that it can be cited independently in the future. Additionally, PLOS ONE offers an option to publish peer-reviewed clinical study protocols. Read more information on sharing protocols at https://plos.org/protocols?utm_medium=editorial-email&utm_source=authorletters&utm_campaign=protocols

# Author comment: Author response to Decision Letter 1

**DOI:** 10.1371/journal.pdig.0001122.r002

13 May 2026

Attachment Submitted filename: REVISED RESPONSE TO REVIEWERS.docx

# Peer review: Decision Letter 1

**DOI:** 10.1371/journal.pdig.0001122.r003

**Authors:** Mengyu Wang, Daphna Idan

6 Jul 2026

Response to ReviewersRevised Manuscript with Track ChangesManuscript**Journal Requirements:** **Additional Editor Comments (if provided):**

**Reviewers' Comments:**

**Comments to the Author**

Reviewer #3: All comments have been addressed

**********

publication criteria?

Reviewer #3: Yes

**********

3. Has the statistical analysis been performed appropriately and rigorously?

Reviewer #3: Yes

**********

4. Have the authors made all data underlying the findings in their manuscript fully available (please refer to the Data Availability Statement at the start of the manuscript PDF file)?

The PLOS Data policy

Reviewer #3: Yes

**********

5. Is the manuscript presented in an intelligible fashion and written in standard English?

Reviewer #3: Yes

**********

Reviewer #3: The authors have substantially improved the manuscript and addressed most of my prior concerns. The main remaining issues are:

1. The Methods section should be revised to ensure that the sex-inclusivity checklist domains match the deposited checklist on OSF. In the manuscript, Domain 5 is described as “considerations for prevention strategies,” whereas the checklist appears to define Domain 5 as “Tone & Language.” This inconsistency should be corrected throughout the Methods and inter-rater reliability description.

2. The interpretation of the low inter-rater reliability observed for male-tailored materials appears speculative. Weighted kappa values ranging from -0.06 to 0.19 indicate poor agreement but do not establish that the disagreement arose from genuine ambiguity in male-specific CVD content. I recommend presenting the reliability findings descriptively only and reserving interpretation for the Discussion section.

3. Consider adding to the Limitations section that only GPT-4 was evaluated in this study. As a result, the extent to which these findings generalize to other contemporary LLMs, model versions, or prompting approaches remains unknown.

**********

what does this mean?). If published, this will include your full peer review and any attached files.

**Do you want your identity to be public for this peer review?**  If you choose “no”, your identity will remain anonymous but your review may still be made public.

For information about this choice, including consent withdrawal, please see our Privacy Policy

Reviewer #3: No

**********

**Figure resubmission:**

**Reproducibility:**

To enhance the reproducibility of your results, we recommend that authors of applicable studies deposit laboratory protocols in protocols.io, where a protocol can be assigned its own identifier (DOI) such that it can be cited independently in the future. Additionally, PLOS ONE offers an option to publish peer-reviewed clinical study protocols. Read more information on sharing protocols at https://plos.org/protocols?utm_medium=editorial-email&utm_source=authorletters&utm_campaign=protocols

# Author comment: Author response to Decision Letter 2

**DOI:** 10.1371/journal.pdig.0001122.r004

8 Jul 2026

Attachment Submitted filename: R2R ROUND 2.docx

# Editor report: Decision Letter 2

**DOI:** 10.1371/journal.pdig.0001122.r005

**Authors:** Mengyu Wang, Daphna Idan

30 Jul 2026

GPT-4 Improves Sex-Specificity in Cardiovascular Patient Education but May Perpetuate Gender Biases: A Mixed-Methods Audit

PDIG-D-25-01045R2

Dear Ms. Khan,

We are pleased to inform you that your manuscript 'GPT-4 Improves Sex-Specificity in Cardiovascular Patient Education but May Perpetuate Gender Biases: A Mixed-Methods Audit' has been provisionally accepted for publication in PLOS Digital Health.

Before your manuscript can be formally accepted you will need to complete some formatting changes, which you will receive in a follow-up email from a member of our team.

Please note that your manuscript will not be scheduled for publication until you have made the required changes, so a swift response is appreciated.

IMPORTANT: The editorial review process is now complete. PLOS will only permit corrections to spelling, formatting or significant scientific errors from this point onwards. Requests for major changes, or any which affect the scientific understanding of your work, will cause delays to the publication date of your manuscript.

If your institution or institutions have a press office, please notify them about your upcoming paper to help maximize its impact. If they'll be preparing press materials, please inform our press team as soon as possible -- no later than 48 hours after receiving the formal acceptance. Your manuscript will remain under strict press embargo until 2 pm Eastern Time on the date of publication. For more information, please contact digitalhealth@plos.org.

Thank you again for supporting Open Access publishing; we are looking forward to publishing your work in PLOS Digital Health.

Best regards,

Daphna Idan, Md mph

Guest Editor

PLOS Digital Health

***********************************************************

**Additional Editor Comments (if provided):**

**Reviewer Comments (if any, and for reference):**
